# Learning While Doing: Community-Engaged Action Research With Age-Friendly Community Leaders

**DOI:** 10.1093/geroni/igaa057.2446

**Published:** 2020-12-16

**Authors:** Althea Pestine-Stevens, Emily Greenfield, Stephanie Firestone

## Abstract

Age-Friendly Community Initiatives (AFCIs) are expanding throughout the United States to make social and built environments within local communities more responsive to population aging. With over 450 initiatives affiliated with the AARP Network of Age-Friendly Communities, Cities, and States (125 of which began in 2019-early 2020), rapid growth on the ground necessitates that theory and research develop alongside practice innovations. This symposium showcases the intersection of cutting-edge scholarship with community-based efforts to generate knowledge of community change processes that is immediately actionable by community leaders. Collectively, these papers emphasize the benefits of action research and developmental evaluation in community gerontology towards building the theories of age-friendly change that will set the stage for outcomes research. The first paper will present on work with 83 AFCIs in rural Maine involving interviews with organizational leaders to inform which types of supports could stimulate age-friendly changes to communities’ built and social environments. The second paper will share a mixed-methods approach used to develop a global toolkit for dementia-friendly communities. The third presenter will describe the collaborative development and utilization of social network analysis to help age-friendly leaders plan their work, while simultaneously advancing research on variation in AFCI implementation. The final paper will present an evaluative framework that identifies roles and outcome measures for collective impact at the intersection of public health and age-friendly communities.

